# Optimizing MR imaging for intraoperative image guidance in sellar pathologies

**DOI:** 10.1007/s11102-020-01035-1

**Published:** 2020-03-13

**Authors:** Alexander Micko, Arthur Hosmann, Wolfgang Marik, Sophie Bartsch, Michael Weber, Engelbert Knosp, Stefan Wolfsberger

**Affiliations:** 1 Department of Neurosurgery, Medical University of Vienna, Waehringer Guertel 18-20, 1097 Vienna, Austria; 2 Department of Biomedical Imaging and Image-guided Therapy, Medical University of Vienna, Vienna, Austria

**Keywords:** Pituitary adenoma, Neuronavigation, VIBE, CISS

## Abstract

**Purpose:**

With the advancement of extended endonasal approaches, the ability to surgically reach parasellar tumor extensions increase. The aim of the study was to propose an optimized imaging protocol for surgical guidance in the cavernous sinus (CS) for proper visualization structures at risk.

**Methods:**

Prospective case control analysis of 20 consecutive pituitary adenoma patients scheduled for endoscopic transnasal surgery. Assessment of the capability of three different MRI sequences (MPRAGE, VIBE, CISS) by 4 investigators to correctly visualize sellar and parasellar structures. Invasiveness and position of the normal pituitary gland were compared with the intraoperative findings.

**Results:**

The consensus between the 4 examiners to achieve the same results for all modalities was 40% for MPRAGE, 70% for VIBE and 60% for CISS sequences (p = 0.155). A consensus of Knosp Grade per patient was 80% for MPRAGE, 100% for VIBE and 90% for CISS (overall kappa 0.60). A higher Knosp Grade was found in MPRAGE sequences compared to the other sequences. Intraoperative status of invasiveness was correctly identified in 12/20 (60%) with MPRAGE, 19/20 (95%) with VIBE and 11/20 (55%) with CISS sequences. The position of the normal pituitary gland was most frequent evaluable in 15/20 (75%) and correctly identified in 12/15 (80%) cases.

**Conclusion:**

Our data showed that VIBE sequences obtain the highest degree of consensus with intraoperative findings of invasiveness and position of the normal pituitary gland. VIBE sequences, due to their high spatial resolution and at the same time fast image acquisition could provide improved imaging for neuronavigation.

## Introduction

Pituitary adenomas are neuroendocrine tumors that show invasive growth into surrounding structures in up to 40% [1–5]. Especially parasellar invasiveness into cavernous sinus (CS) structures is still one of the most important prognostic factors for dismal outcome [6–9].

With the advancement of extended endonasal approaches (EEA), the ability to surgically reach parasellar tumor extensions increases. Hence, there is a demand for proper visualization of the neurovascular CS structures at risk such as cranial nerves and arteries and their relation to parasellar pituitary adenoma components.

The imaging modality of choice for soft tissue structures is magnetic resonance imaging (MRI). Currently, contrast enhanced T1-weighted MPRAGE MRI is widely used for delineation of pituitary gland and adenoma tissue as well as for the identification of the intracavernous internal carotid artery (ICA), because of its short acquisition time and isovoxel capability.

Neuronavigation systems, which by reconstruction of isovoxel image data provide the surgeon with multiplanar views for preoperative planning and intraoperative orientation, have found wide acceptance for use in endoscopic transsphenoidal surgery [10–20], especially for EEA. However, MPRAGE sequences only provide limited visualization detail of the adenoma extensions into and their relation to the neurovascular structures within the CS.

With advancements in MRI technology, sequences have been developed that can readily identify the tumor extension into the CS as well as the CS structures in a preoperative planning setting [21, 22]. Two sequences have turned out to be the most useful:

**Fat sat VIBE** (volumetric interpolated breath-hold examination) images are fat-suppressed sequences that permit short acquisition times with high-resolution and therefore allow volumetric acquisition with an isotropic matrix. This allows high-resolution multiplanar reconstruction giving not only parenchymal but also vascular information at the same time through the application of contrast agent. VIBE sequences can therefore be used for high spatial resolution and improved soft tissue contrast imaging of the pituitary [23, 24].

**CISS** (constructive interference in steady state) sequences on the other hand provide better visualization of the cranial nerves and cisternal spaces due to the ability to accentuate T2-weighted values [25]. Especially by the usage of gadolinium based contrast agents, superior delineation of CS nerves in the intracavernous segment can be achieved [26]. CISS has thereby been shown to have a predictive value of postoperative visual outcomes and adenoma pathology in retrospective studies [27–29].

The aim of the study was to propose an optimized imaging protocol for surgical guidance in the CS by intraoperatively comparing tumor extension and neurovascular structures visualized on different MRI sequences with the concurrent surgical findings.

## Methods

Prospective case control analysis of 20 consecutive pituitary adenoma patients scheduled for endoscopic transnasal surgery at our institution in 2019. We assessed the capability of three different MRI sequences (MPRAGE, VIBE, CISS) to correctly visualize cavernous sinus neurovascular structures and the grade of parasellar tumor extension compared with the intraoperative view.

The following parameters were evaluated: Parasellar tumor extension (according to the revised Knosp classification [5]), surgical invasiveness, identification and position of the normal pituitary gland, the optic chiasm, and the oculomotor nerve within the cavernous sinus.

Histopathological analysis according to the 2017 WHO classification of pituitary adenomas was performed as in the routine postoperative setting [30].

This study was approved by the hospital’s ethics committee (EC Nr. 1549/2019) and was performed in accordance with the principles of the Declaration of Helsinki. Prior to enrollment into the trial, participants gave their written informed consent.

### Magnet resonance imaging parameters

For this study, our routine preoperative MRI protocol for pituitary image guidance (MPRAGE, MRA—time of flight) was extended with VIBE and CISS sequences (additional 9 min scanning time). All examinations were performed on a 3T magnetic resonance scanner (3 T Vida, Siemens) using a 64-channel head and neck coil. Exclusion criteria were: claustrophobia, metal devices in or on the subject’s body which contradict a 3T examination. (Table 1)

**Table 1:** Patient characteristics

	Patient cohort	
	n (IQR)	%
Number of patients	20	
Age (median, years)	38 (28–59)	
Gender		
Female	13	65
Male	7	35
Primary/re-operation	18/2	
Tumor size*, mm (median max. diameter)	18.5 (11–24)	
Micro-/macroadenoma	4/16	
Functional classification		
Functional	9	45
Non-functional	11	55
WHO 2017 classification		
Somatotroph	1	5
Lactotroph	6	30
Thyrotroph	1	5
Corticotroph	1	5
Gonadotroph	6	30
Null cell	4	20
Plurihormonal	1	5

*IQR* interquartile range

*Mean diameter of MPRAGE/VIBE/CISS

*MPRAGE*: coronary slices, isovoxel imaging (0.9 mm voxel size), TR: 2300 ms, TE: 2.32 ms, Matrix: 256, Flip angle: 8°; FoV: 240 mm, slice thickness: 0.9 mm, number of slices: 180, acquisition time: 5:21 min;

*VIBE*: coronary slices, fat saturation, isovoxel imaging (0.7 mm voxel size), TR: 7.01 ms, TE: 2.58 ms, Matrix: 288, Flip angle: 15°; FoV: 210 mm, slice thickness: 0.7 mm, number of slices: 144, acquisition time: 3:17 min;

*CISS*: coronary slices, 0.5 mm voxel size, TR: 8.33 ms, TE: 3.89 ms, Matrix: 256, Flip angle: 50°; FoV: 140 mm, slice thickness: 0.5 mm, number of slices: 80, acquisition time: 6:51 min.

### Neuronavigation

This study was based on our multimodality navigation protocol published in 2019 that includes computed tomography (CT) for solid bone structures and fine paranasal sinus structures [31], MRA-TOF for vascular anatomy, and standard T1-weighted CE MRI (MPRAGE) for pituitary and CS structures. The latter was always found insufficient to visualize CS structures in detail.

**Fig. 1:**
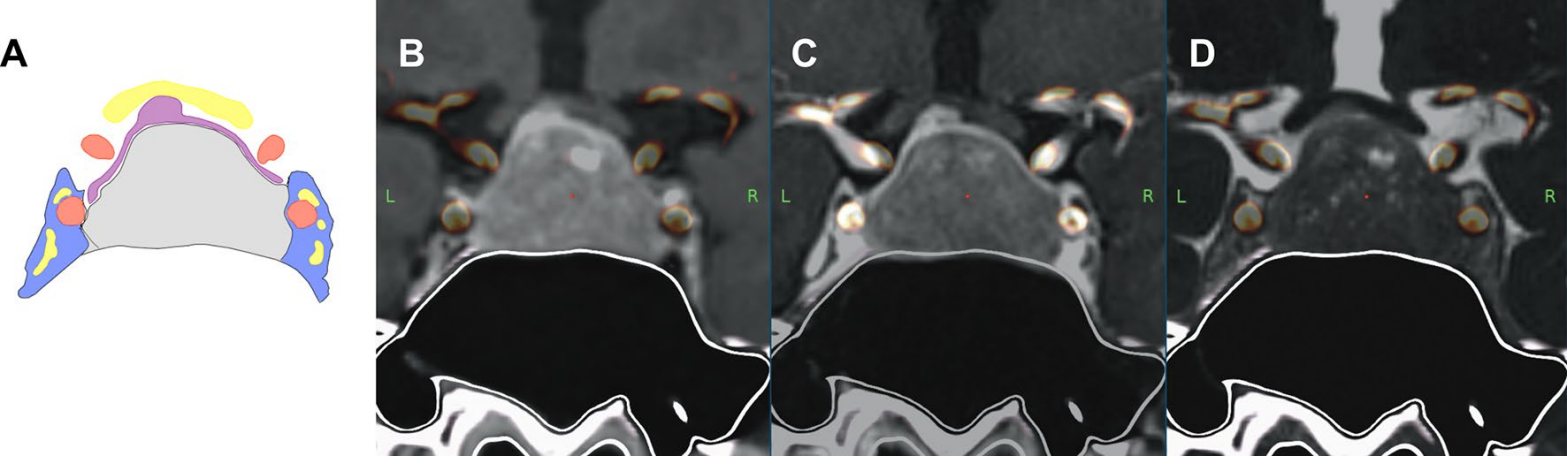
Multimodality Imaging, combination of MR, 2x CT (1 for solid bone structures, 1 for visualization of fine paranasal structures), MR angiogram (time-of-flight). **a** Schematic drawing: grey = pituitary adenoma, purple = normal pituitary gland, red = internal carotid artery (ICA), yellow = cranial nerves, blue = cavernous sinus (CS). **b** Multimodality imaging, MPRAGE as MR sequence. **c** Multimodality imaging, VIBE as MR sequence. **d** Multimodality imaging, CISS as MR sequence

**Fig. 2:**
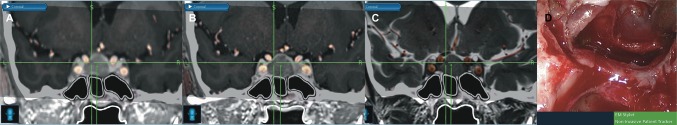
Intraoperative Neuronavigation, correlation with anatomic structures. **a** Multimodality imaging, MPRAGE as MR sequence. **b** Multimodality imaging, VIBE as MR sequence. **c** Multimodality imaging, CISS as MR sequence. **d** Intraoperative image; an electromagnetic stylet was placed in a suction device. Green line with dot…. planned trajectory at the center of the pituitary adenoma. Green crosshair… depiction of the location of the tip of the electromagnetic stylet within the suction device

The ability of the three MRI sequences (MPRAGE, VIBE or CISS) to correctly visualize the anatomy of the CS as part of the multimodality MRI imaging protocol was assessed by four investigators, two neuroradiologists (S.B. and W.M.) and two neurosurgeons (A.H. and A.M.) blinded to the intraoperative results. (Fig. 1) The following parameters were assessed on neuronavigation systems (Stealth Station S7, Medtronic, USA): Identification and position of the normal pituitary gland, visibility of the oculomotor nerves, parasellar tumor extension (graded according to the revised Knosp classification [5]) and surgical invasiveness. Consensus results of investigators were then compared to the ground truth of intraoperative findings. (Fig. 2)

### Statistical evaluation

The data are presented as median and interquartile range (IQR) for continuous variables and as frequencies for categorical variables. Agreement upon the 4 reviewers had to be at least 3/4 to conclude a consensus.

The consensus for each assessed parameter was used to compare the MRI sequences (MPRAGE, VIBE or CISS) among each other. Additionally, the consensus of invasiveness and identification and position of the normal pituitary gland was correlated with the results of intraoperative findings. The probability of a consensus between the 4 examiners was carried out using the Cochran Q test. The agreement of the raters per examined modality and sequence was calculated using Fleiss Kappa.

A p-value < 0.05 will be considered significant. For statistical analyses SPSS® version 24.0 software (SPSS Inc., Chicago, IL, USA) was used.

## Results

### Patient characteristics

Median age at the time of operation was 38 years (IQR 28-59). Functioning adenomas represented 9/20 (45%) of patients, whereas 11/20 (55%) were non-functioning adenomas. Within the study cohort 4/20 (20%) were microadenomas and 16/20 (80%) were macroadenomas. The maximal tumor diameter, calculated as a mean of MPRAGE/VIBE/CISS sequences, was median 18.5 mm (IQR 11-24 mm). For further patient characteristics, see Table 1.

### Rater consensus

The consensus between the 4 examiners for all parameters assessed (grade of parasellar tumor extension according to the revised Knosp Classification; invasiveness; identification and position of the normal pituitary gland; visibility of the optic chiasm; visibility of the oculomotor nerves) was 40% for MPRAGE, 70% for VIBE and 60% for CISS sequences (p = 0.155).

Only the position of the normal pituitary gland showed significant differences of examiners consensus between the MR sequences (p = 0.045), in all other examined parameters no significant difference was present.

Parasellar tumor extension as evaluated by the revised Knosp classification showed a complete consensus of examiners in 28/40 (70%) (left and right CS compartment) independent of the examined MR sequence.

A consensus of Knosp grade per patient was 80% for MPRAGE, 100% for VIBE and 90% for CISS (overall kappa 0.60). A higher Knosp grade was found in MPRAGE sequences in 6 cases, compared to VIBE and CISS sequences. (Table 2)

**Table 2:** Knosp Classification between the MR sequences (40 cavernous sinus compartments)

	Knosp 0	Knosp 1	Knosp 2	Knosp 3A	Knosp 3B	Knosp 4
MPRAGE*	18	8	5	1	–	4
VIBE	24	8	7	–	–	1
CISS*	21	7	2	6	–	–

*…in 4 cavernous sinus compartments no consensus was reached

Intraoperative invasiveness was found in 4/20 (20%) of cases. A false positive result turned out in 3 MPRAGE, 1 VIBE and 3 CISS cases; a false negative result turned out in 2 CISS cases.

#### Pituitary gland location

The normal pituitary gland was found to be located in 13/20 (65%) cases lateral and in 7/20 (35%) cases superior to the tumor mass. A false lateral (instead of intraoperative superior) position was suspected in 2 MPRAGE, 3 of VIBE and 3 of CISS cases. A false superior (instead of intraoperative lateral) position was suspected in 2 CISS cases.

Cranial nerves: The optic chiasm by consensus could be detected in 13/17 (76%) of MPRAGE, 16/18 (89%) of VIBE and 17/19 (89%) of CISS cases.

The oculomotor nerve within the cavernous sinus could be detected by consensus in 13/19 (68%) of MPRAGE, 11/15 (73%) of VIBE and 13/15 (87%) CISS cases. (Table 3)

**Table 3:** Evaluation of invasiveness, position of the pituitary gland, optic chiasm and oculomotor nerve

	Invasiveness		Normal gland position		Optic chiasm detected	Oculomotor nerve detected
	Consensus	Accuracy	Consensus	Accuracy		
	n/n (%)	n/n (%)	n/n (%)	n/n (%)	n/n (%)	n/n (%)
MPRAGE	15/20 (75)	12/20 (60)	8/20 (40)	6/20 (30)	13/17* (76)	13/19* (68)
VIBE	20/20 (100)	19/20 (95)	15/20 (75)	12/20 (60)	16/18* (89)	11/15* (73)
CISS	16/20 (80)	11/20 (55)	11/20 (55)	6/20 (30)	17/19* (89)	13/15* (87)

*…in the missing cases no consensus was reached between examiners

## Discussion

The MR sequences most widely used for intraoperative guidance are conventional T1-weighted CE MPRAGE sequences [20, 32]. However, newer MR sequences such as VIBE and CISS have been found to identify intra- and parasellar structures to a higher detail. According to our data of multimodality neuronavigation imaging, MPRAGE sequences tend to overestimate the parasellar extension of the tumor mass. VIBE sequences showed the highest degree of consensus with intraoperative findings of invasiveness and position of the normal pituitary gland. CISS sequences could identify the oculomotor nerve in most of the cases.

### MR sequences for visualization of sellar and parasellar structures

Especially in cases of large tumors markedly displacing the optic chiasm, the normal gland tissue and neurovascular structures within the cavernous sinus, a proper correlation of intraoperative and neuronavigation images is crucial. Sequences that are able to establish high spatial resolution and therefore the possibility to better distinguish contrast difference have been investigated [22, 23]. VIBE sequences have been found to reach a high spatial resolution and therefore improve the visualization of soft tissue. CISS or FIESTA (Fast Imaging Employing Steady-state Acquisition, GE Healthcare equivalent of CISS) have a high fluid signal and high spatial resolution.

Xie et al. [33] first presented a case series of 7 patients where they used FIESTA sequences in the preoperative planning to approach sellar and parasellar lesions and found to properly visualize vascular and neural structures.

### Parasellar adenoma extension

Knosp grading was found variable between the MR sequences. Our results of pituitary adenomas are in accordance with Lang et al. [21] that MPRAGE sequences have been found to overestimate the parasellar tumor extension. CISS sequences might misinterpret the parasellar extension as they are prone to artifacts [34]. In contrast to the results of Lang et al. we found VIBE sequences to correlate with the highest degree (95%) to intraoperatively identified tumor invasiveness by direct endoscopic visualization. VIBE may facilitate the depiction of invasive tumor components through a higher grade of visualization of enhancing structures within the CS. Therefore, a distinction between pituitary adenoma tissue and CS structures may be more accurate in VIBE than in MPRAGE or CISS sequences.

Position of the pituitary gland: Preoperative localization of the normal pituitary gland is crucial to prevent inadvertent surgical damage and consecutive hormonal insufficiency. To define the position of the pituitary gland, Davis et al. compared conventional T1-weighted CE sequences versus VIBE sequences in 32 patients, and found in more than 50% of cases that investigators rated delineation of the gland more accurately with VIBE [23]. In our prospective analysis investigators reached the highest grade of consensus on VIBE sequences (15/20) and the gland was correctly identified in 12/15 cases (80%). In comparison to conventional T1-weighted CE sequences (MPRAGE) a worse rate of consensus was reached (8/20, 6/8 correctly identified) in CISS sequences (11/20 consensus), and the position of the gland could only be correctly identified in 6/11 cases (54%).

#### Cranial nerves

CISS sequences provide a good delineation of the optic pathway and cranial nerves relative to the tumor mass because of a low signal intensity and proper distinction of cerebrospinal fluid [27, 28]. In case of tumor involvement, the visualization of cranial nerves within the CS with these sequences has been found to be depended which nerve is involved. Amemiya et al. were able to visualize the oculomotor nerve in all cases whereas the abducent nerve was only seen in half of their cases [35].

Although CISS sequences have a striking advantage in visualization of cranial nerves, the possible overestimation of parasellar invasive growth and disadvantage of failing to correctly delineate the position of remaining normal gland tissue are drawbacks for using primarily this sequence for neuronavigation. However, due to the possibility to mark the cranial nerves as region of interests, this information can be used in a multimodality imaging setting as an addition.

#### Microadenomas

Detection of microadenomas by VIBE sequences has been found to improve the detection from 60–80% compared with conventional T1-weighted CE MRI sequences [36–39].

Examining previously MR-negative patients with Cushing’s disease, Grober et al. [24] found that SGE (spoiled-gradient echo 3D T1 imaging; GE Healthcare equivalent of VIBE) is superior to conventional T1-weighted CE MRI sequences to identify corticotroph microadenomas. From our series, we cannot confirm these results, as only 20% (4/20) were microadenomas and no corticotroph adenoma was included. However, we found VIBE sequences to better delineate the pituitary gland details.

### Limitations

Due to the limited number of patients included in this prospective trial, no significant difference of investigators consensus between the three MR sequences could be reached. However, to the best of our knowledge this is the first study investigating MPRAGE/VIBE/CISS sequences in a multimodality neuronavigation setting.

Although VIBE sequences have been found to correlate with the highest degree with intraoperative findings such as invasiveness and position of the remaining pituitary gland, this sequence has a high susceptibility to motions causing artifacts.

## Conclusions

With the advancement of extended endonasal approaches the demand for proper visualization of sellar and parasellar structures at risk via neuronavigation images is necessary. In this prospective trial, our data showed that VIBE sequences obtain the highest degree of consensus with intraoperative findings of invasiveness and position of the normal pituitary gland. Therefore, VIBE sequences due to their high spatial resolution and at the same time fast image acquisition could provide improved preoperative images in an isovoxel protocol for neuronavigation.

However, additional CISS sequences could be necessary to identify the oculomotor nerve in pituitary adenomas that show an extensive parasellar growth.
